# Mental health first aid training in a workplace setting: A randomized controlled trial [ISRCTN13249129]

**DOI:** 10.1186/1471-244X-4-23

**Published:** 2004-08-15

**Authors:** Betty A Kitchener, Anthony F Jorm

**Affiliations:** 1 Depression & Anxiety Consumer Research Unit, Centre for Mental Health Research, Australian National University, Canberra, Australia; 2 Centre for Mental Health Research, Australian National University, Canberra, Australia

## Abstract

**Background:**

The Mental Health First Aid training course was favorably evaluated in an uncontrolled trial in 2002 showing improvements in participants' mental health literacy, including knowledge, stigmatizing attitudes, confidence and help provided to others. This article reports the first randomized controlled trial of this course.

**Methods:**

Data are reported on 301 participants randomized to either participate immediately in a course or to be wait-listed for 5 months before undertaking the training. The participants were employees in two large government departments in Canberra, Australia, where the courses were conducted during participants' work time. Data were analyzed according to an intention-to-treat approach.

**Results:**

The trial found a number of benefits from this training course, including greater confidence in providing help to others, greater likelihood of advising people to seek professional help, improved concordance with health professionals about treatments, and decreased stigmatizing attitudes. An additional unexpected but exciting finding was an improvement in the mental health of the participants themselves.

**Conclusions:**

The Mental Health First Aid training has shown itself to be not only an effective way to improve participants' mental health literacy but also to improve their own mental health. It is a course that has high applicability across the community.

## Background

In 2000 we developed a Mental Health First Aid course in response to the findings of two large national mental health surveys in Australia [1,2]. These findings included a high prevalence rate of mental health problems (approximately 20% of adults in any one year), the poor mental health literacy of members of the Australian public (poor recognition and knowledge of symptoms and causes of mental health problems, where to seek help and what are the most effective treatments) and the widespread stigma towards people with mental health problems. Regular first aid courses are recognised as improving the public's giving of initial and appropriate help at medical emergencies but, unfortunately, most of these courses do not include mental health problems.

The Mental Health First Aid course consists of three weekly sessions of three hours each. The content covers helping people in mental health crises and/or in the early stages of mental health problems. The crisis situations covered included suicidal thoughts and behavior, acute stress reaction, panic attacks and acute psychotic behavior. The mental health problems discussed included depressive, anxiety and psychotic disorders. The co-morbidity with substance use disorders is also covered. Participants learn the symptoms of these disorders, possible risk factors, where and how to get help and evidence-based effective help.

The initial evaluation trial of the Mental Health First Aid course was an uncontrolled one with 210 members of the public with pre, post and 6-month follow-up. This trial showed that participants improved: their recognition of mental disorders, their beliefs about what treatments were helpful, attitudes towards people with mental illness, the amount of help provided to people with mental health problems, and their confidence in providing help to these people [3].

The next step in our evaluation of this course was to conduct a randomised trial involving a wait-list control group. The present article reports this study, which was carried out in a workplace setting.

## Methods

### Participants

Eligible participants (approximately 4800) were all Canberra-based employees of two Australian government departments: Health and Ageing, and Family and Community Services. The trial was advertised to staff by email. Participants had to agree to be randomly assigned to receive the training in either Month 1 or Month 6. Training was delivered and data collected at the worksite during office hours.

### Interventions

The course content has been described in the Background and previously [3] and further details can be found at the Mental Health First Aid website [4]. The training followed set lesson plans and all participants were given a Mental Health First Aid Manual to keep [5]. Training was administered at the worksite in classes of 6–18 participants. Participants did not necessarily stay in the same class, but moved between classes to complete the course as necessitated by their work schedule. One instructor carried out all the training. She is the developer of the Mental Health First Aid course and had trained over 1000 people before the start of the trial. Participants received training either immediately (June) or after a five-month delay (November). Those who received training immediately constituted the intervention group and the wait-listed group was the control. To monitor whether the intervention was actually received, an attendance roll was kept for each class.

### Objectives

The main objective was to assess whether Mental Health First Aid training improved mental health literacy and helping skills relative to a wait-list control. A secondary objective was to assess any benefits to the participants' own mental health.

### Outcomes

Outcomes were measured in the month before intervention (the pre-test assessment) and in the fifth month after intervention (the follow-up assessment). The intervention group received training in Month 1 (immediately after pre-test) and the wait-list control group received training in Month 6 (immediately after the follow-up).

All outcomes were measured by self-completed questionnaires based on the ones used in the uncontrolled trial of Mental Health First Aid [3]. The pre-test questionnaire (see Additional File 1) covered the following: socio-demographic characteristics of the participant, why they were interested in doing the course, history of mental health problems in participant or family, confidence in providing help, contact with people who have mental health problems in previous 6 months and help offered, recognition of a disorder in vignettes describing a person with depression and one with schizophrenia, belief about the helpfulness of various interventions for the persons described, a social distance scale to assess stigmatizing attitudes [7], and whether the participant or a family member or friend had ever had a problem like the one in the vignette.

To score the items on beliefs about treatment, a scale was created showing the extent to which participants agreed with health professionals about which interventions would be useful. For depression, there is a professional consensus that GPs, psychiatrists, clinical psychologists, antidepressants, counseling and cognitive-behavior therapy are helpful [6]. Thus, participants received a score from 0 to 6 according to the number of these interventions endorsed as helpful and this was converted into a percentage. For schizophrenia, there is a professional consensus that GPs, psychiatrists, clinical psychologists, antipsychotics and admission to a ward are helpful for schizophrenia [6]. "Helpful" ratings were summed to give a score from 0 to 5 and converted to a percentage.

The questionnaire ended with the SF-12, which provided scales assessing the participant's mental and physical health [8]. These scales were scored using Andrews' [9] integer scorer.

The follow-up questionnaire was the same as the pre-test questionnaire except that it omitted the sociodemographic questions and asked about contact with anyone with a mental health problem over the 5 months since the last questionnaire (rather than 6 months).

The questionnaires were sent out via internal departmental mail by a human resources staff member in each place of employment. The questionnaires were completed anonymously with only an ID number and posted back to the researchers at the Centre for Mental Health Research. The IDs of any non-responders were sent back to the human resources staff member who sent out a reminder. The researchers were never told the names of individual respondents and the human resources staff member in the place of employment never saw any completed questionnaires or individually identifiable data.

### Sample size

The study was planned to have a sample of 300. The sample size was determined by practical constraints: when it was convenient to run classes that fitted the employees' work schedule and the workload on the instructor. It was determined that this sample size had excellent power to detect medium effect sizes for both continuous and dichotomous outcomes [10]. The trial was originally planned to involve only one workplace, but was extended to a second one because the number of participants recruited was smaller than expected. The lower recruitment appeared to be due to the requirement that participants agree to random assignment to training at either of two periods.

### Randomization and blinding

A staff member in the human resources section of the place of employment kept a list of participants' names and ID numbers. The researchers only had access to the IDs. One of the researchers (Jorm) randomly assigned participants to training or control groups by ID number using the Random Integers option at the  website [11]. After recruitment, participants were assigned an ID by the staff member in human resources. These staff assigned participants to groups based on the randomized IDs provided to them. Random allocation occurred only after all participants within a place of employment were recruited and assigned ID numbers. The instructor (Kitchener) provided the human resources staff member with the names of attendees to check that participation was as allocated. Blinding was not possible with the Mental Health First Aid intervention.

### Ethics

Ethical approval for the study was given by the Australian National University Human Research Ethics Committee.

### Statistical methods

Repeated measures analysis of variance was used to analyze continuous measures, with two groups (intervention and control) and two time points (pre-test and follow-up). The principal interest was in the group × time interaction effect. Logistic regression was used to analyze change in dichotomous measures, with group and pre-test score as the predictors and follow-up score as the outcome. Place of employment was also investigated to see if there was a difference in the effects of training. However, no interaction effects involving place of employment were found, so this variable was dropped from all analyses reported below.

The analysis was carried out according to intention-to-treat principles, so that all persons who completed a pre-test questionnaire were included, even if they subsequently dropped out. In such cases, the pre-test score was substituted for the missing value, so that no improvement was assumed.

## Results

### Recruitment

An email inviting participation was sent to all staff of the relevant departments based in Canberra. The email was sent out in May 2002 for the Department of Health and Ageing and March 2003 for the Department of Family and Community Services. In order to participate, staff had to send back a consent form and fill out a pre-test questionnaire before the start of classes.

### Participant flow

**Figure 1:**
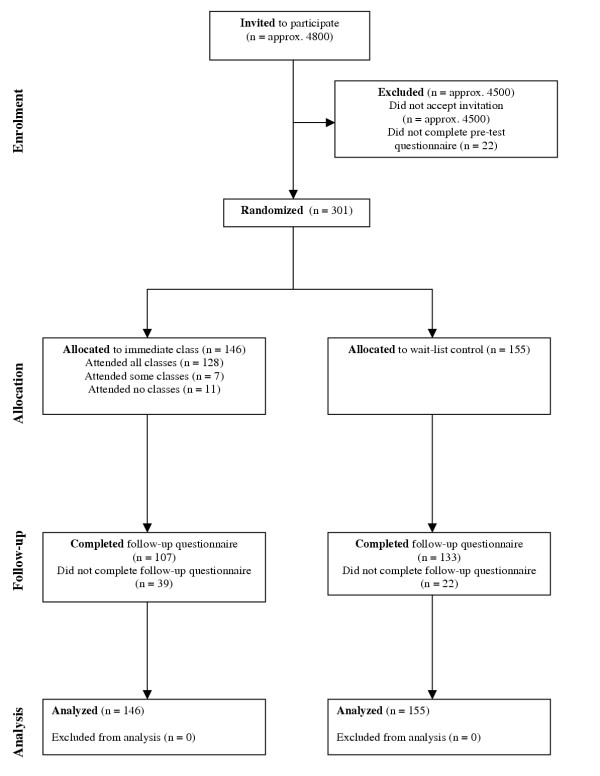
Flow diagram showing progress through the phases of the trial.

Figure 1 shows the flow of participants at each stage of the trial. There were two deviations from plan. Firstly, 18 of the 146 participants (12.3%) assigned to receive Mental Health First Aid training did not complete the whole course. Secondly, 39 out of 146 participants (26.7%) in the intervention group did not complete follow-up questionnaires, compared to only 22 out of 155 (14.2%) in the control group.

### Participants' characteristics

In terms of sociodemographic characteristics, 78.1% of the participants were female, 49.2% were aged 18–39 years, 50.2% were aged 40–59 and 0.7% aged 60+ years. There were 60.6% with a university degree, 1.3% were aboriginal and 8.6% did not have English as their first language. 13.0% described themselves as mental health consumers, 9.6% as carers for a person with a mental health problem, and 6.3% as health service providers. When asked their reason for doing the course, 27.2% cited reasons relating to their workplace, 11.7% reasons relating to family or close friends, 4.9% reasons relating to their own mental health status, 20.5% cited duty as a citizen, 29% said they were just interested, and 6.7% wanted more accurate or updated information on mental health. 165 (54.8%) of the participants worked at the Department of Health and Ageing and 136 (45.2%) at the Department of Family and Community Services.

### Numbers analyzed

The data were analyzed according to intention-to-treat principles, so that all persons who completed a pre-test questionnaire were included, even if they subsequently dropped out. For every analysis, there were 146 participants analyzed in the intervention group and 155 in the control group.

### Perception of mental health problem in self or family

Participants were asked about whether they themselves had ever experienced a mental health problem or whether anyone in their family had. Table 1 shows that around half reported having personally experienced a mental health problem and around three-quarters reported that a family member had a mental health problem. However, participating in the Mental Health First Aid course did not affect these variables.

**Table 1:** Percent reporting history of mental health problem in self or family.

Mental health problems in:	MHFA group	Control group	P-value for group × time interaction
Self			.577
Pre-test	60.0%	49.7%	
Follow-up	65.5%	55.6%	
Change (95% CI)	5.5% (0.5 to 10.6)	5.9% (0.6 to 11.1)	
Family			.849
Pre-test	74.5%	73.0%	
Follow-up	77.2%	75.7%	
Change (95% CI)	2.8% (-3.9 to 9.4)	2.6% (-3.5 to 8.7)	

### Recognition of disorder in vignette

Table 2 shows the percentage who correctly recognized the disorders in the vignettes. For the schizophrenia vignette, mention of either "schizophrenia" or "psychosis" was considered correct. The table also shows the percentage who got both vignettes correct. Although there tended to be greater improvement in recognition in the group receiving Mental Health First Aid, there were no significant differences from the control group.

**Table 2:** Percent correctly recognizing the disorder in a vignette.

Type of vignette	MHFA group	Control group	P-value for group × time interaction
Depression			.091
Pre-test	90.2%	87.7%	
Follow-up	95.8%	90.3%	
Change (95% CI)	5.6% (0.5 to 10.7)	2.6% (-2.8 to 8.0)	
Schizophrenia			.083
Pre-test	74.6%	83.9%	
Follow-up	82.6%	81.9%	
Change (95% CI)	8.0% (1.5 to 14.4)	-2.0% (-6.8 to 2.8)	
Both vignettes			.189
Pre-test	70.6%	76.5%	
Follow-up	80.2%	77.8%	
Change (95% CI)	9.6% (2.8 to 16.4)	1.3% (-5.2 to 7.9)	

### Beliefs about treatments

Table 3 shows the data on whether beliefs about treatments became more concordant with those of health professionals. There was significantly greater improvement in concordance in the Mental Health First Aid group when both depression and schizophrenia were considered together. However, the trends failed to reach significance at the .05 level when the disorders were considered separately.

**Table 3:** Changes in beliefs about treatment and in social distance.

Scale	MHFA group	Control group	P-value for group × time interaction
Beliefs about treatment for depression			.062
Pre-test mean (SD)	82.10 (17.27)	83.00 (18.95)	
Follow-up mean (SD)	86.29 (18.30)	83.42 (18.48)	
Change (95% CI)	4.19 (1.18 to 7.20)	0.42 (-2.20 to 3.04)	
Beliefs about treatment for schizophrenia			.096
Pre-test mean (SD)	84.28 (19.33)	88.21 (16.76)	
Follow-up mean (SD)	87.41 (18.26)	88.41 (16.11)	
Change (95% CI)	3.13 (0.30 to 5.96)	0.20 (-1.87 to 2.27)	
Beliefs about treatment for both disorders			.036
Pre-test mean (SD)	83.28 (16.65)	85.51 (15.05)	
Follow-up mean (SD)	86.98 (16.78)	85.89 (14.42)	
Change (95% CI)	3.70 (1.16 to 6.24)	0.38 (-1.46 to 2.23)	
Social distance from person with depression			.005
Pre-test mean (SD)	8.74 (2.80)	8.63 (2.63)	
Follow-up mean (SD)	7.86 (2.50)	8.46 (2.54)	
Change (95% CI)	-0.88 (-1.23 to -0.53)	-0.18 (-0.51 to 0.16)	
Social distance from person with schizophrenia			.211
Pre-test mean (SD)	12.12 (3.53)	12.13 (3.50)	
Follow-up mean (SD)	11.27 (3.50)	11.62 (3.35)	
Change (95% CI)	-0.84 (-1.23 to -0.46)	-0.51 (-0.87 to -0.15)	
Social distance from both			.020
Pre-test mean (SD)	20.88 (5.79)	20.79 (5.53)	
Follow-up mean (SD)	19.14 (5.43)	20.07 (5.30)	
Change (95% CI)	-1.73 (-2.37 to -1.10)	-0.72 (-1.29 to -0.14)	

### Social distance

Table 3 shows data on social distance from the person in each vignette. There was greater improvement in social distance in the Mental Health First Aid group overall, but when the two vignettes were examined separately, this improvement was confined to the depression vignette.

### Help provided to others

Table 4 shows data on confidence in providing help and actual help provided to others in the period before completing the questionnaire. Confidence improved more in the Mental Health First Aid group. There was no change in the percentage who reported contact with anyone with a mental health problem or in the percentage reporting giving "some" or "a lot" of help. However, while the control group showed a decline in the percentage advising professional help, the Mental Health First Aid group did not, leading to a significant difference between groups.

**Table 4:** Changes in confidence and help provided to others.

Outcome	MHFA group	Control group	P-value for group × time interaction
% Feeling confident in helping someone ("moderately", "quite a lot" or "extremely")			.001
Pre-test	54.5%	49.7%	
Follow-up	74.5%	57.4%	
Change (95% CI)	20.0% (12.6 to 27.4)	7.7% (1.3 to 14.1)	
% Had contact with anyone with mental health problem			.157
Pre-test	71.5%	70.8%	
Follow-up	72.9%	65.6%	
Change (95% CI)	1.4% (-6.9 to 9.6)	-5.2% (-13.5 to 3.1)	
% Provided help ("some" or "a lot")			.525
Pre-test	37.0%	37.5%	
Follow-up	39.0%	36.2%	
Change (95% CI)	2.0% (-5.5 to 9.6)	-1.3% (-9.6 to 6.9)	
% Advised professional help			.007
Pre-test	28.1%	27.1%	
Follow-up	29.4%	16.8%	
Change (95% CI)	1.4% (-6.8 to 9.5)	-10.3% (-18.0 to -2.6)	

### Participants' mental health

Table 5 shows changes in the mental and physical health of participants. The Mental Health First Aid group showed significantly greater improvement in mental health. No difference between groups was found in physical health, but none was expected. The physical health scale is included in the table only to show the specificity of the effect on mental health.

**Table 5:** Changes in mental and physical health.

Scale	MHFA group	Control group	P-value for group × time interaction
Mental health			.035
Pre-test mean (SD)	45.43 (11.40)	45.40 (10.17)	
Follow-up mean (SD)	47.48 (11.11)	45.11 (11.25)	
Change (95% CI)	2.06 (0.39 to 3.72)	-0.29 (-1.72 to 1.14)	
Physical health			.506
Pre-test mean (SD)	51.38 (7.97)	51.97 (8.11)	
Follow-up mean (SD)	50.74 (8.14)	51.90 (8.68)	
Change (95% CI)	-0.64 (-1.80 to 0.53)	-0.07 (-1.29 to 1.16)	

### Adverse events

Given that an educational intervention was evaluated with a non-clinical sample, there was no justification for a systematic inquiry into adverse events. Informally, no adverse events were reported.

## Discussion

This trial has found a number of benefits from Mental Health First Aid training. Relative to the control group, the intervention group showed greater confidence in providing help to others, greater likelihood of advising people to seek professional help, improved concordance with health professionals in beliefs about treatment, decreased social distance from people suffering from depression, and improved mental health of the participants themselves. Recognition of disorders in vignettes did not improve, but there was a very high recognition at pre-test, limiting the scope for improvement.

A potential criticism of Mental Health First Aid training is that it will lead to excessive labeling of life problems as mental disorders by members of the public. To check this possibility we asked participants about mental health problems in themselves and family members. Although a high prevalence rate was reported, we found that the course had no effect on these rates.

A surprising effect was that the course improved the participants' scores on the SF-12 mental health scale. We included this scale to explore whether there was any impact on mental health, but did not have any strong expectation that it would. The course is not aimed at the participants' own mental health and does not include any therapy. Furthermore, only 5% of participants cited their own mental health as a reason for doing the course. Nevertheless, the participants' mean score on the mental health scale was around half a standard deviation below Australian population norms [9], showing that some were having on-going problems. The cause of the improvement in mental health is not clear. It is unlikely to be a placebo effect because the course gave no expectation of personal change in mental health and only a small percentage did the course for their own benefit. Furthermore, there was no corresponding change on the SF-12 physical health scale. We speculate that the evidence-based information given in the course allowed participants to take action to benefit their own mental health. A similar therapeutic effect has recently been reported from a trial of a web site giving evidence-based information on depression [12].

The data analysis involved a conservative intention-to-treat strategy in which participants who failed to complete the whole course were included and those who failed to respond to the follow-up questionnaire were assumed to show no change. A particular limitation in the present study is that participants in the intervention group showed a poorer response to the follow-up questionnaire than controls. The reason for this poorer response is unknown, but we believe it occurred because the intervention group had already received the course and had nothing to gain by filling out a further questionnaire. By contrast, the controls were still waiting to receive their training and may have believed that filling out the questionnaire would assist this. Whatever the reason, the poorer response in the intervention group meant that more of them were assumed to show no change, thus minimizing any benefits of the training. It is likely that the true effects of Mental Health First Aid training are greater than the present data indicate.

The present trial evaluates efficacy rather than effectiveness. The trial was carried out in a workplace setting with well-educated employees who were allowed to do the course during working hours. There was only one instructor, who was the developer of the Mental Health First Aid course, limiting the generalizability of the findings to other instructors. Further research is needed to evaluate the course as taught by other instructors in more typical settings. We are currently engaged in an effectiveness trial with members of the public in a large rural area, with local health service staff trained to run the courses.

The Mental Health First Aid training evaluated in this trial was 9 hours long. Based on feedback from participants that the course needed to be longer, we now routinely run the course over 12 hours. This longer course expands on each of the topics covered, especially substance use disorders. Whether this longer course has additional benefits remains to be evaluated. However, our expectation is that it would produce greater effects on beliefs about treatment, confidence in providing help and actual help to provided to others.

## Conclusions

Mental Health First Aid training appears to be effective in improving some aspects of mental health literacy, confidence in providing help to others, and the type of help provided. The training also benefits the mental health of participants. The course is highly acceptable in a workplace setting and could be widely applied. Over 100 Mental Health First Aid instructors have now been trained and the course is available throughout much of Australia and in Scotland, Hong Kong and New York State, USA. Dissemination in other localities is planned in the near future.

## Competing interests

The authors were the developers of the Mental Health First Aid course.

## Authors' contributions

BAK co-designed the study and the evaluation questionnaire, taught the Mental Health First Aid courses, and co-wrote the manuscript.

AFJ co-designed the study and the evaluation questionnaire, analyzed the data, and co-wrote the manuscript. Both authors read and approved the final manuscript.

## Pre-publication history

The pre-publication history for this paper can be accessed here:



## Supplementary Material

Additional File 1 Pre-test questionnaire. questions used in pre-test questionnaire Click here for file
