# Supplementary material for: Mental health first aid training in a workplace setting: A randomized controlled trial [ISRCTN13249129]
Source: BMC Psychiatry. 2004 Aug 15;4:23. doi: 10.1186/1471-244X-4-23 (PMC514553; doi:10.1186/1471-244X-4-23)
Supplement: Additional File 1 — Pre-test questionnaire. questions used in pre-test questionnaire [file 1471-244X-4-23-S1.doc]

1. What is your sex?

Male ⬜ 1

Female ⬜ 2

1. How old are you?

years

1. What is the highest level of schooling that you completed?

Some primary ⬜ 1

All of primary ⬜ 2

Some secondary ⬜ 3

Three/four years of secondary (intermediate or

school certificate level) ⬜ 4

Five/six years of secondary (leaving or

higher school certificate level) ⬜ 5

4. What is the highest level of post secondary/tertiary education you completed?

Trade/apprenticeship ⬜ 1

Other certificate ⬜ 2

Associate or undergraduate diploma ⬜ 3

Bachelor’s degree ⬜ 4

Higher degree ⬜ 5

Other ⬜ 6

None ⬜ 7

1. Are you presently studying for any of the following?

Trade/apprenticeship ⬜ 1

Other certificate ⬜ 2

Associate or undergraduate diploma ⬜ 3

Bachelor’s degree ⬜ 4

Higher degree ⬜ 5

Other ⬜ 6

1. Are you:

Aboriginal ⬜ 1

Torres Strait Islander ⬜ 2

Both ⬜ 3

Neither ⬜ 4

1. Do you speak a language other than English as your first language at home?

Yes ⬜ 1

No ⬜ 2

1. Do you belong to any of the following groups?

(Tick as many boxes as apply)

Mental health consumer ⬜ 1

Carer of person with a mental health problem ⬜ 2

Health Service Provider ⬜ 3

Specify area/discipline ________________________

Law enforcement officer ⬜ 4

Educator ⬜ 5

1. Why are you interested in doing this course?

____________________________________________________________________________________________________________________________________________________

10. We would like to know a bit about your awareness and experience of mental health problems in your everyday life.

1. Have you yourself ever experienced a mental health problem?

Yes ⬜ 1

No ⬜ 2

1. Has anyone in your family ever experienced a mental health problem?

Yes ⬜ 1

No ⬜ 2

1. How confident do you feel in helping someone with a mental health problem?

Not at all ⬜ 1

A little bit ⬜ 2

Moderately ⬜ 3

Quite a bit ⬜ 4

Extremely ⬜ 5

1. In the last **6 months** have you had contact with anyone with a mental health problem?

Yes ⬜ 1

No ⬜ 2

Don’t know ⬜ 3

1. If “yes” How many people?

Have you offered any help?

Not at all ⬜ 1

A little ⬜ 2

Some ⬜ 3

A lot ⬜ 4

What type of help? ____________________________________________________________________________________________________________________________________________________

____________________________________________________________________________________________________________________________________________________

_______________________________________________________________________________________________________________

The following section concerns a hypothetical person called Mary. The description below outlines how she has been recently.

**Mary is 30 years old. She has been feeling unusually sad and miserable for the last few weeks. Even though she is tired all the time, she has trouble sleeping nearly every night. Mary doesn’t feel like eating and has lost weight. She can’t keep her mind on her work and puts off making any decisions. Even day-to-day tasks seem too much for her. This has come to the attention of Mary’s boss who is concerned about her lowered productivity.**

1. From the information given, what, if anything, is wrong with Mary?

_____________________________________

_____________________________________

_____________________________________

_____________________________________

1. Do you think Mary needs professional help?

Yes ⬜ 1

No ⬜ 2

1. *If Mary were to seek help from any of the following people, is it likely to be helpful, harmful or neither for her (Tick one response for each line)*
2. A typical GP or family doctor

Helpful ⬜ 1

Neither ⬜ 2

Harmful ⬜ 3

1. A chemist or pharmacist

Helpful ⬜ 1

Neither ⬜ 2

Harmful ⬜ 3

1. A counsellor

Helpful ⬜ 1

Neither ⬜ 2

Harmful ⬜ 3

1. A social worker

Helpful ⬜ 1

Neither ⬜ 2

Harmful ⬜ 3

1. Telephone counselling service, e.g. Lifeline

Helpful ⬜ 1

Neither ⬜ 2

Harmful ⬜ 3

1. A psychiatrist

Helpful ⬜ 1

Neither ⬜ 2

Harmful ⬜ 3

1. A clinical psychologist

Helpful ⬜ 1

Neither ⬜ 2

Harmful ⬜ 3

1. Help from her close family

Helpful ⬜ 1

Neither ⬜ 2

Harmful ⬜ 3

1. Help from some close friends

Helpful ⬜ 1

Neither ⬜ 2

Harmful ⬜ 3

1. A naturopath or a herbalist

Helpful ⬜ 1

Neither ⬜ 2

Harmful ⬜ 3

1. The clergy, a minister or a priest

Helpful ⬜ 1

Neither ⬜ 2

Harmful ⬜ 3

1. Mary tries to deal with her problems on her own

Helpful ⬜ 1

Neither ⬜ 2

Harmful ⬜ 3

1. *If Mary were to take one of the following medications, is it likely to be helpful, harmful or neither for her? (Tick one response for each line)*
2. Vitamins and minerals

Helpful ⬜ 1

Neither ⬜ 2

Harmful ⬜ 3

1. St John’s wort

Helpful ⬜ 1

Neither ⬜ 2

Harmful ⬜ 3

1. Pain relievers such as aspirin, codeine or panadol

Helpful ⬜ 1

Neither ⬜ 2

Harmful ⬜ 3

1. Antidepressants

Helpful ⬜ 1

Neither ⬜ 2

Harmful ⬜ 3

1. Antibiotics

Helpful ⬜ 1

Neither ⬜ 2

Harmful ⬜ 3

1. Sleeping pills

Helpful ⬜ 1

Neither ⬜ 2

Harmful ⬜ 3

1. Anti-psychotics

Helpful ⬜ 1

Neither ⬜ 2

Harmful ⬜ 3

1. Tranquillisers such as valium

Helpful ⬜ 1

Neither ⬜ 2

Harmful ⬜ 3

1. *If Mary were to undertake any of the following, is it likely to be helpful, harmful or neither for her? (Tick one response for each line)*
2. Becoming more physically active such as playing more sport, or doing a lot more walking or gardening

Helpful ⬜ 1

Neither ⬜ 2

Harmful ⬜ 3

1. Read about people with similar problems and how they have dealt with them

Helpful ⬜ 1

Neither ⬜ 2

Harmful ⬜ 3

1. Getting out and about more

Helpful ⬜ 1

Neither ⬜ 2

Harmful ⬜ 3

1. Courses on relaxation, stress management, meditation or yoga

Helpful ⬜ 1

Neither ⬜ 2

Harmful ⬜ 3

1. Cutting out alcohol altogether

Helpful ⬜ 1

Neither ⬜ 2

Harmful ⬜ 3

1. Counselling

Helpful ⬜ 1

Neither ⬜ 2

Harmful ⬜ 3

1. Cognitive-behavioural therapy

Helpful ⬜ 1

Neither ⬜ 2

Harmful ⬜ 3

1. Psychotherapy

Helpful ⬜ 1

Neither ⬜ 2

Harmful ⬜ 3

1. Hypnosis

Helpful ⬜ 1

Neither ⬜ 2

Harmful ⬜ 3

1. Admission to a psychiatric ward of a hospital

Helpful ⬜ 1

Neither ⬜ 2

Harmful ⬜ 3

1. Electroconvulsive therapy (ECT)

Helpful ⬜ 1

Neither ⬜ 2

Harmful ⬜ 3

1. Having an occasional alcoholic drink to relax

Helpful ⬜ 1

Neither ⬜ 2

Harmful ⬜ 3

1. A special diet or avoiding certain foods

Helpful ⬜ 1

Neither ⬜ 2

Harmful ⬜ 3

1. *How willing would you be to :*
2. Move next door to Mary

Definitely willing ⬜ 1

Probably willing ⬜ 2

Probably unwilling ⬜ 3

Definitely unwilling ⬜ 4

1. Spend an evening socializing with Mary

Definitely willing ⬜ 1

Probably willing ⬜ 2

Probably unwilling ⬜ 3

Definitely unwilling ⬜ 4

1. Make friends with Mary

Definitely willing ⬜ 1

Probably willing ⬜ 2

Probably unwilling ⬜ 3

Definitely unwilling ⬜ 4

1. Have Mary start working closely with you on a job

Definitely willing ⬜ 1

Probably willing ⬜ 2

Probably unwilling ⬜ 3

Definitely unwilling ⬜ 4

1. Have Mary marry into your family

Definitely willing ⬜ 1

Probably willing ⬜ 2

Probably unwilling ⬜ 3

Definitely unwilling ⬜ 4

1. Have you ever had a problem similar to Mary’s?

Yes ⬜ 1

No ⬜ 2

18. Has anyone in your family or close circle of friends ever had a problem similar to Mary’s?

Yes ⬜ 1

No ⬜ 2

The following section concerns a hypothetical person called John. The description below outlines how he has been recently.

**John is 24 and lives at home with his parents. He has had a few temporary jobs since finishing school but is now unemployed. Over the last 6 months he has stopped seeing his friends, and has begun locking himself in his bedroom and refusing to eat with the family or to have a bath. His parents also hear him walking about in his bedroom at night while they are in bed. Even though they know he is alone, they have heard him shouting and arguing as if someone else is there. When they try to encourage him to do more things, he whispers that he won’t leave home because he is being spied upon by the neighbour. They realise he is not taking drugs because he never sees anyone or goes anywhere.**

1. From the information given, what, if anything, is wrong with John?

____________________________________________________________________________________________________________________________________________________

1. Do you think John needs professional help?

Yes ⬜ 1

No ⬜ 2

1. *If John were to seek help from any of the following people, is it likely to be helpful, harmful or neither for him (Tick one response for each line)*
2. A typical GP or family doctor

Helpful ⬜ 1

Neither ⬜ 2

Harmful ⬜ 3

1. A chemist or pharmacist

Helpful ⬜ 1

Neither ⬜ 2

Harmful ⬜ 3

1. A counsellor

Helpful ⬜ 1

Neither ⬜ 2

Harmful ⬜ 3

1. A social worker

Helpful ⬜ 1

Neither ⬜ 2

Harmful ⬜ 3

1. Telephone counselling service, e.g. Lifeline

Helpful ⬜ 1

Neither ⬜ 2

Harmful ⬜ 3

1. A psychiatrist

Helpful ⬜ 1

Neither ⬜ 2

Harmful ⬜ 3

1. A clinical psychologist

Helpful ⬜ 1

Neither ⬜ 2

Harmful ⬜ 3

1. Help from his close family

Helpful ⬜ 1

Neither ⬜ 2

Harmful ⬜ 3

1. Help from some close friends

Helpful ⬜ 1

Neither ⬜ 2

Harmful ⬜ 3

1. A naturopath or a herbalist

Helpful ⬜ 1

Neither ⬜ 2

Harmful ⬜ 3

1. The clergy, a minister or a priest

Helpful ⬜ 1

Neither ⬜ 2

Harmful ⬜ 3

1. John tries to deal with his problems on his own

Helpful ⬜ 1

Neither ⬜ 2

Harmful ⬜ 3

1. *If John were to take one of the following medications, is it likely to be helpful, harmful or neither for him? (Tick one response for each line)*
2. Vitamins and minerals

Helpful ⬜ 1

Neither ⬜ 2

Harmful ⬜ 3

1. St John’s wort

Helpful ⬜ 1

Neither ⬜ 2

Harmful ⬜ 3

1. Pain relievers such as aspirin, codeine or panadol

Helpful ⬜ 1

Neither ⬜ 2

Harmful ⬜ 3

1. Antidepressants

Helpful ⬜ 1

Neither ⬜ 2

Harmful ⬜ 3

1. Antibiotics

Helpful ⬜ 1

Neither ⬜ 2

Harmful ⬜ 3

1. Sleeping pills

Helpful ⬜ 1

Neither ⬜ 2

Harmful ⬜ 3

1. Anti-psychotics

Helpful ⬜ 1

Neither ⬜ 2

Harmful ⬜ 3

1. Tranquillisers such as valium

Helpful ⬜ 1

Neither ⬜ 2

Harmful ⬜ 3

1. *If John were to undertake any of the following, is it likely to be helpful, harmful or neither for him? (Tick one response for each line)*
2. Becoming more physically active such as playing more sport, or doing a lot more walking or gardening

Helpful ⬜ 1

Neither ⬜ 2

Harmful ⬜ 3

1. Read about people with similar problems and how they have dealt with them

Helpful ⬜ 1

Neither ⬜ 2

Harmful ⬜ 3

1. Getting out and about more

Helpful ⬜ 1

Neither ⬜ 2

Harmful ⬜ 3

1. Courses on relaxation, stress management, meditation or yoga

Helpful ⬜ 1

Neither ⬜ 2

Harmful ⬜ 3

1. Cutting out alcohol altogether

Helpful ⬜ 1

Neither ⬜ 2

Harmful ⬜ 3

1. Counselling

Helpful ⬜ 1

Neither ⬜ 2

Harmful ⬜ 3

1. Cognitive-behavioural therapy

Helpful ⬜ 1

Neither ⬜ 2

Harmful ⬜ 3

1. Psychotherapy

Helpful ⬜ 1

Neither ⬜ 2

Harmful ⬜ 3

1. Hypnosis

Helpful ⬜ 1

Neither ⬜ 2

Harmful ⬜ 3

1. Admission to a psychiatric ward of a hospital

Helpful ⬜ 1

Neither ⬜ 2

Harmful ⬜ 3

1. Electroconvulsive therapy (ECT)

Helpful ⬜ 1

Neither ⬜ 2

Harmful ⬜ 3

1. Having an occasional alcoholic drink to relax

Helpful ⬜ 1

Neither ⬜ 2

Harmful ⬜ 3

1. A special diet or avoiding certain foods

Helpful ⬜ 1

Neither ⬜ 2

Harmful ⬜ 3

1. *How willing would you be to :*
2. Move next door to John

Definitely willing ⬜ 1

Probably willing ⬜ 2

Probably unwilling ⬜ 3

Definitely unwilling ⬜ 4

1. Spend an evening socializing with John

Definitely willing ⬜ 1

Probably willing ⬜ 2

Probably unwilling ⬜ 3

Definitely unwilling ⬜ 4

1. Make friends with John

Definitely willing ⬜ 1

Probably willing ⬜ 2

Probably unwilling ⬜ 3

Definitely unwilling ⬜ 4

1. Have John start working closely with you on a job

Definitely willing ⬜ 1

Probably willing ⬜ 2

Probably unwilling ⬜ 3

Definitely unwilling ⬜ 4

1. Have John marry into your family

Definitely willing ⬜ 1

Probably willing ⬜ 2

Probably unwilling ⬜ 3

Definitely unwilling ⬜ 4

25. Have you ever had a problem similar to John’s?

Yes ⬜ 1

No ⬜ 2

26. Has anyone in your family or close circle of friends ever had a problem similar to John’s?

Yes ⬜ 1

No ⬜ 2

The next few questions ask for your views about your health, how you feel and how well you are able to do your usual activities on a typical day. If you are unsure about how to answer a question, please give the best answer you can.

1. In general, would you say your health is

Excellent ⬜ 1

Very good ⬜ 2

Good ⬜ 3

Fair ⬜ 4

Poor ⬜ 5

The following questions are about activities you might do during a typical day. Does your health now limit you in these activities? If so, how much?

1. Moderate activities, such as moving a table, pushing a vacuum cleaner, bowling or playing golf?

Yes – limited a lot ⬜ 1

Yes – limited a little ⬜ 2

No – not limited at all ⬜ 3

1. Climbing *several* flights of stairs?

Yes – limited a lot ⬜ 1

Yes – limited a little ⬜ 2

No – not limited at all ⬜ 3

During the *past 4 weeks*, have you had any of the following problems with your work or other regular daily activities as a result of *your physical health*?

1. Have you *accomplished less* than you would like as a result of *your physical health*?

Yes ⬜ 1

No ⬜ 2

1. Were you limited in the *kind* of work or other activities as a result of *your physical health*?

Yes ⬜ 1

No ⬜ 2

During the *past 4 weeks*, have you had any of the following problems with your work or other regular daily activities as a result of any *emotional problems (such as feeling depressed or anxious)*?

32. Have you *accomplished less* than you would like as a result of *any emotional problems*?

Yes ⬜ 1

No ⬜ 2

1. Did you not do work or other activities as *carefully* as usual as a result of any *emotional problems*?

Yes ⬜ 1

No ⬜ 2

1. During the *past 4 weeks*, how much did *pain* interfere with your normal work (including both work outside the home and housework)?

Not at all ⬜ 1

A little bit ⬜ 2

Moderately ⬜ 3

Quite a bit ⬜ 4

Extremely ⬜ 5

The next few questions are about how you feel and how things have been with you *during the past 4 weeks*. For each question, please give the one answer that comes closest to the way you have been feeling.

1. How much of the time during the past 4 weeks *have you felt calm and peaceful*?

All of the time ⬜ 1

Most of the time ⬜ 2

A good bit of the time ⬜ 3

Some of the time ⬜ 4

A little of the time ⬜ 5

None of the time ⬜ 6

36. How much of the time during the past 4 weeks *did you have a lot of energy*?

All of the time ⬜ 1

Most of the time ⬜ 2

A good bit of the time ⬜ 3

Some of the time ⬜ 4

A little of the time ⬜ 5

None of the time ⬜ 6

1. How much of the time during the past 4 weeks *have you felt down*?

All of the time ⬜ 1

Most of the time ⬜ 2

A good bit of the time ⬜ 3

Some of the time ⬜ 4

A little of the time ⬜ 5

None of the time ⬜ 6

1. How much of the time during the past 4 weeks has your *physical health or emotional problems interfered with your social activities* (like visiting with friends, relatives, etc)?

All of the time ⬜ 1

Most of the time ⬜ 2

Some of the time ⬜ 3

A little of the time ⬜ 4

None of the time ⬜ 5

You have finished! Thank you.

**Please return the questionnaire in the envelope provided.**
